# Chronic Methamphetamine Effects on Brain Structure and Function in Rats

**DOI:** 10.1371/journal.pone.0155457

**Published:** 2016-06-08

**Authors:** Panayotis K. Thanos, Ronald Kim, Foteini Delis, Mala Ananth, George Chachati, Mark J. Rocco, Ihssan Masad, Jose A. Muniz, Samuel C. Grant, Mark S. Gold, Jean Lud Cadet, Nora D. Volkow

**Affiliations:** 1 Behavioral Neuropharmacology and Neuroimaging Laboratory on Addictions, Research Institute on Addictions, University at Buffalo, Buffalo, NY, United States of America; 2 Department of Psychology, UNC Chapel Hill, Chapel Hill, NC, United States of America; 3 Department of Pharmacology, School of Medicine, University of Ioannina, Ioannina, Greece; 4 Department of Neuroscience, Stony Brook University, Stony Brook, NY, United States of America; 5 National High Magnetic Field Laboratory, Tallahassee, FL, United States of America; 6 Washington University School of Medicine, Department of Psychiatry, St. Louis, MO, United States of America; 7 Molecular Neuropsychiatry Research Branch, NIDA, NIH, Department of Health and Human Services, Baltimore, MD, United States of America; 8 Laboratory of Neuroimaging, NIAAA, NIH, Department of Health and Human Services, Bethesda, MD, United States of America; Hudson Institute, AUSTRALIA

## Abstract

Methamphetamine (MA) addiction is a growing epidemic worldwide. Chronic MA use has been shown to lead to neurotoxicity in rodents and humans. Magnetic resonance imaging (MRI) studies in MA users have shown enlarged striatal volumes and positron emission tomography (PET) studies have shown decreased brain glucose metabolism (BGluM) in the striatum of detoxified MA users. The present study examines structural changes of the brain, observes microglial activation, and assesses changes in brain function, in response to chronic MA treatment. Rats were randomly split into three distinct treatment groups and treated daily for four months, via i.p. injection, with saline (controls), or low dose (LD) MA (4 mg/kg), or high dose (HD) MA (8 mg/kg). Sixteen weeks into the treatment period, rats were injected with a glucose analog, [^18^F] fluorodeoxyglucose (FDG), and their brains were scanned with micro-PET to assess regional BGluM. At the end of MA treatment, magnetic resonance imaging at 21T was performed on perfused rats to determine regional brain volume and in vitro [^3^H]PK 11195 autoradiography was performed on fresh-frozen brain tissue to measure microglia activation. When compared with controls, chronic HD MA-treated rats had enlarged striatal volumes and increases in [^3^H]PK 11195 binding in striatum, the nucleus accumbens, frontal cortical areas, the rhinal cortices, and the cerebellar nuclei. FDG microPET imaging showed that LD MA-treated rats had higher BGluM in insular and somatosensory cortices, face sensory nucleus of the thalamus, and brainstem reticular formation, while HD MA-treated rats had higher BGluM in primary and higher order somatosensory and the retrosplenial cortices, compared with controls. HD and LD MA-treated rats had lower BGluM in the tail of the striatum, rhinal cortex, and subiculum and HD MA also had lower BGluM in hippocampus than controls. These results corroborate clinical findings and help further examine the mechanisms behind MA-induced neurotoxicity.

## Introduction

Methamphetamine (MA) is a widely abused drug with devastating health effects [1]. MA increases extracellular concentrations of dopamine (DA), norepinephrine (NE) and serotonin (5HT) by acting on the transporter of each neurotransmitter [2–4] and by reversing neurotransmitter transport direction [5]. Chronic abuse of MA has been associated with damage to DA and 5HT terminals [5–7].

Additionally MA is rapidly taken by various organs in the body, including the lungs, brain, liver, pancreas, stomach, and kidneys, where it clears slowly [8], which could explain the association between MA and pulmonary hypertension [9] and kidney damage [10] among others. However, most MA studies have focused on the central nervous system. Studies in humans and non-human primates using magnetic resonance imaging (MRI) have shown structural abnormalities in the brain of MA users, including lower gray matter volumes [11], increased white matter volumes [11], enlarged striatal volumes [12–17] and larger volumes of the parietal cortex [14]. This increased brain volume in MA users has been hypothesized to reflect inflammatory changes in these brain regions, including microglial activation, and has been positively correlated with deterioration of performance in reversal learning [17]. Still, volumetric increases in the striatum of MA users have also been positively correlated with novelty seeking [15] and with improved cognitive performance [13, 16], which suggests that increased striatal volume after MA use may also reflect compensatory changes in response to MA-induced neurotoxicity.

Using positron emission tomography (PET) and the glucose analog fluorodeoxyglucose (FDG), regional brain glucose metabolism (BGluM) has been assessed in MA users. In detoxified MA users, metabolic activity was increased in the parietal cortex and decreased in the thalamus and striatum [18, 19], although some recovery of function was seen in the thalamus after protracted abstinence [19]. Other studies have reported increased metabolism in the parietal cortex [20], increased metabolism in the cingulate, amygdala, ventral striatum, and cerebellum, but decreased metabolism in the insular and orbitofrontal area in abstinent MA users [21]. In addition PET studies have reported a downregulation of DA transporters (DAT) [22] and of DA D2 receptors in the striatum of MA abusers [23] with evidence of some recovery in DAT levels after protracted detoxification [24, 25]. Finally PET studies using [11C]PK 11195, which serves as a marker of microglia activation have also provided evidence of neuroinflammatory changes in the brain of MA users [26].

Together, these studies show that chronic MA use can lead to structural and functional brain deficits, although the possibility of pre-existing vulnerabilities in the brain of human MA users cannot be excluded. To further characterize the effects of long term MA use, the present study examined in rodents, the structural changes using magnetic resonance imaging (MRI), functional changes using PET to measure regional BGluM, and microglial activation with in vitro [^3^H]PK 11195 autoradiography. We hypothesized that chronic MA treatment would result in structural and functional deficits throughout the brain and that these effects would be potentiated in regions linked to the dopaminergic system, including the striatum and the nucleus accumbens. Furthermore, [^3^H]PK 11195 autoradiography would allow us to assess if MA-induced structural and functional changes in the brain were associated with neuroinflammation.

## Methods and Materials

### Animals

Sprague Dawley (SD) rats were purchased from Taconic farms (NY). All rats were male and 4 weeks old at the start of the study. Animals were individually housed in clear plexi-glass cages, with wire covers under standard laboratory conditions (22±2°C, 50±10% relative humidity), and a reverse12h/12h light/dark cycle, with lights on at 8 P.M. and off at 8 A.M. All experimental sessions were conducted during the rats’ dark cycle. Food and water were available *ad-libitum*, except the night before μPET scans when rats were food deprived, similar to previous FDG micro positron emission tomography (μPET) studies [27]. All experiments were conducted in conformity with the National Academy of Sciences Guide for the Care and Use of Laboratory Animals (NAS, 1996) and the University of Buffalo IACUC protocol RIA13095Y.

### Drugs

MA hydrochloride was purchased from Sigma-Aldrich (St. Louis, MO). MA was prepared by dissolving MA hydrochloride in saline solution to produce concentrations of 4 mg/kg and 8 mg/kg. After a one week acclimation period to the animal facility, rats began MA treatment. Rats were randomly assigned to receive vehicle (saline), or low dose (LD) 4mg/kg MA, or high dose (HD) 8 mg/kg MA, via daily intraperitoneal injections for four months (1 ml/kg body weight).

### Procedures

#### Structural magnetic resonance imaging

At the end of MA treatment, rats (n = 6 vehicle, n = 5 LD, n = 8 HD) were transcardially perfused with 4% paraformaldehyde and the heads were scanned on a 21.1T Biospin Advance scanner. T2-weighted MR images were generated with the following parameters: TE = 7.5 ms, TR = 150 ms, FOV = 3.40 cm x 3.12 cm x 3.00 cm, voxel size = 0.08 mm x 0.08 mm x 0.08 mm, scan time 12 hrs. Brains scans were aligned to a common space with rigid transformations using RView, (http://colin-studholme.net [28]) and were manually segmented with the use of the modeling and visualization package Amira 4.1 (Visage Imaging Inc, Andover, MA). Segmentations were guided by the rat brain stereotaxic atlas by Paxinos and Watson [29]. Volumes are reported in mm^3^.

#### [^3^H]PK 11195 autoradiography

Following the last day of treatment, rats were anesthetized using isoflurane and decapitated. The brains were rapidly removed, flash-frozen in 2-methylbutane, and stored at -80°C until use (n = 5 / group). Cryostat sections (14 μm thick) were cut, mounted on slides, and stored tightly sealed at -80°C in the presence of desiccant, until the day of the binding experiment.

[^3^H]PK 11195 binding was carried out according to previously established protocol [30, 31]. Sections were pre-incubated for 15 min in 50 mM Tris-HCl buffer (pH 7.4) at room temperature. Sections were then incubated in pre-incubation buffer with the addition of 0.8 nM [^3^H]PK 11195 (85.7 Ci/mmol, PerkinElmer Inc.) for 30 min at room temperature. Non-specific binding was determined on consecutive sections in the presence of excess 20 μM unlabelled PK 11195. At the end of the incubation, sections were washed twice for 6 minutes in ice-cold 50 mM Tris HCL buffer (pH 7.4) and then dipped in ice-cold distilled water.

After binding, all sections were dried under a stream of cool air and exposed onto Kodax BioMax MR Film, alongside calibrated tritium standards (American Radiolabeled Chemicals, St. Louis, MO). After 8 weeks of exposure, the films were developed in Kodax D-19 developer, dried and scanned as a TIFF image. All regions of interest were quantified using the calibrated standard curves and the Image J software (NIH). Regions of interest (ROI) selected include major areas of the cerebral cortex: prelimbic/infralimbic, cingulate, retrosplenial, insular, rhinal, motor (M1 & M2), piriform, somatosensory, auditory, and visual. Sub-cortical areas selected for analysis include: nucleus accumbens, amygdala, striatum, cerebellum (cortex and nuclei), colliculi, hippocampus, hypothalamus, periaqueductal gray, septum, substantia nigra, and thalamus.

#### Brain Glucose Metabolism using [18F] FDG and μPET

**Fig 1:**
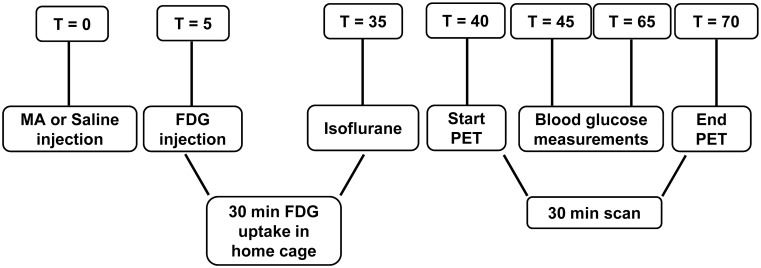
Outline of [18F] FDG μPET experiment. Five minutes following MA or vehicle injection, rats were injected with FDG. After a 30 minute uptake in the home cage, rats were anesthetized and scanned for 30 minutes. T: time (min); MA: methamphetamine; FDG: [18F]deoxyglucose; PET: positron emission tomography.

BGluM was assessed 16 weeks into the treatment period (n = 7 vehicle; n = 9 LD; n = 6 HD), using [18F] FDG and μPET. Briefly (see Fig 1 for timeline), 5 minutes following MA or saline (for vehicle treatment group) injections, rats were injected i.p. with [^18^F] FDG (~750 μCi ± 250 μCi). After a 30 minute awake uptake period in the home cage, rats were anesthetized with isoflurane and scanned for 30 minutes in a μPET R4 scanner (transaxial resolution = 2.0 mm full width at half maximum, field of view = 11.5 mm; Concorde CTI Siemens, Knoxville, TN).

Image analysis was performed as previously described [32]. Briefly, images were reconstructed using a MAP algorithm with 15 iterations, 0.01 smoothing value and a 256 x 256 resolution. After reconstruction, voxel size was x = 2.0, y = 2.0, z = 2.0 mm. All images were spatially normalized and co-registered to the Schweinhardt MRI template (Schweinhardt et al. 2003) using the PMOD software (PMOD Technologies, Zurich, Switzerland). Using the statistical parametric mapping software (SPM 8), images were then smoothed (4 mm Gaussian) and a one-way Analysis of Variance (ANOVA) was run to assess significant differences in metabolic activity between treatment groups. Comparisons between groups were made at Ke = 50 voxels and a = 0.01. These numbers correspond to the minimum number of voxels compared between treatment groups that show significant differences in BGluM with an alpha level set at 0.01.

Brain regions that showed significant differences were overlaid onto the Schweinhardt MRI atlas [33] in Paxinos space, and assessed using the stereotaxic atlas by Paxinos and Watson [29]. Increased BGluM was defined as greater metabolism in MA treated rats (either LD or HD) when compared to vehicle treated rats. Decreased BGluM was defined as greater metabolism in vehicle treated rats when compared with MA (either LD or HD) treated rats.

### Statistics

MRI measures were analyzed with one-way Analysis of Variance (ANOVA), with treatment as between-subjects factor. Analysis of Co-Variance (ANCOVA) with total brain volume as co-factor was also performed. When appropriate, Bonferroni post-hoc test was applied to assess the significant pairwise differences. Auroradiographic [^3^H]PK 11195 measures were analyzed with one-way ANOVA, followed by Holm-Sidak post hoc comparisons when appropriate. Level of significance was set at p<0.05.

## Results

### MRI

**Fig 2:**
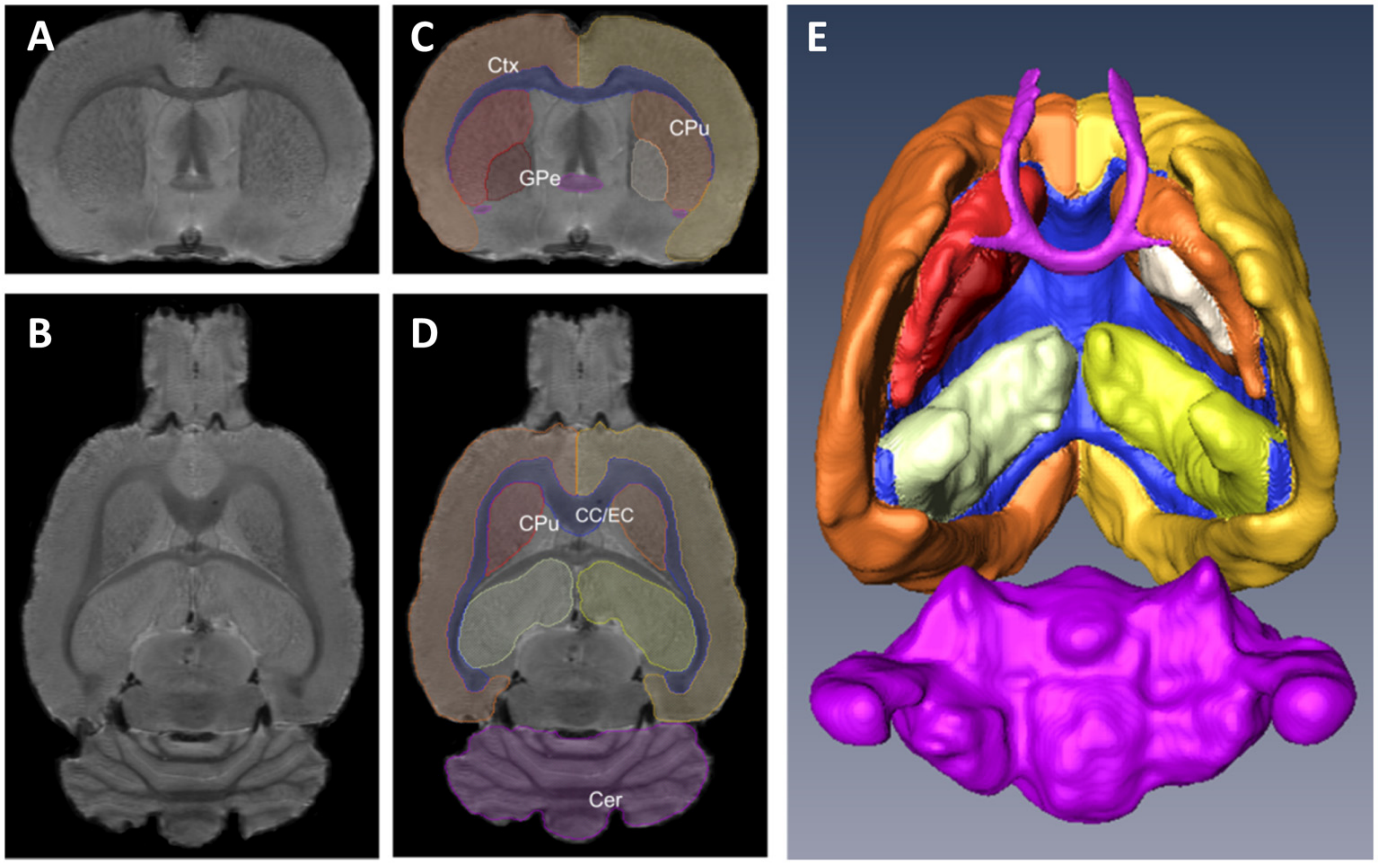
Rat brain MRI slices and three-dimensional reconstruction. A-B Coronal (A) and horizontal (B) brain MRI slices from a rat brain. C-D Outlined regions of interest from the same slices. E Three-dimensional reconstruction of the segmented regions of interest; ventral view. Ctx: cerebral cortex, CPu: caudate-putamen (striatum), GPe: globus pallidus, CC/EC: corpus callosum/external capsule, Cer: cerebellum.

**Fig 3:**
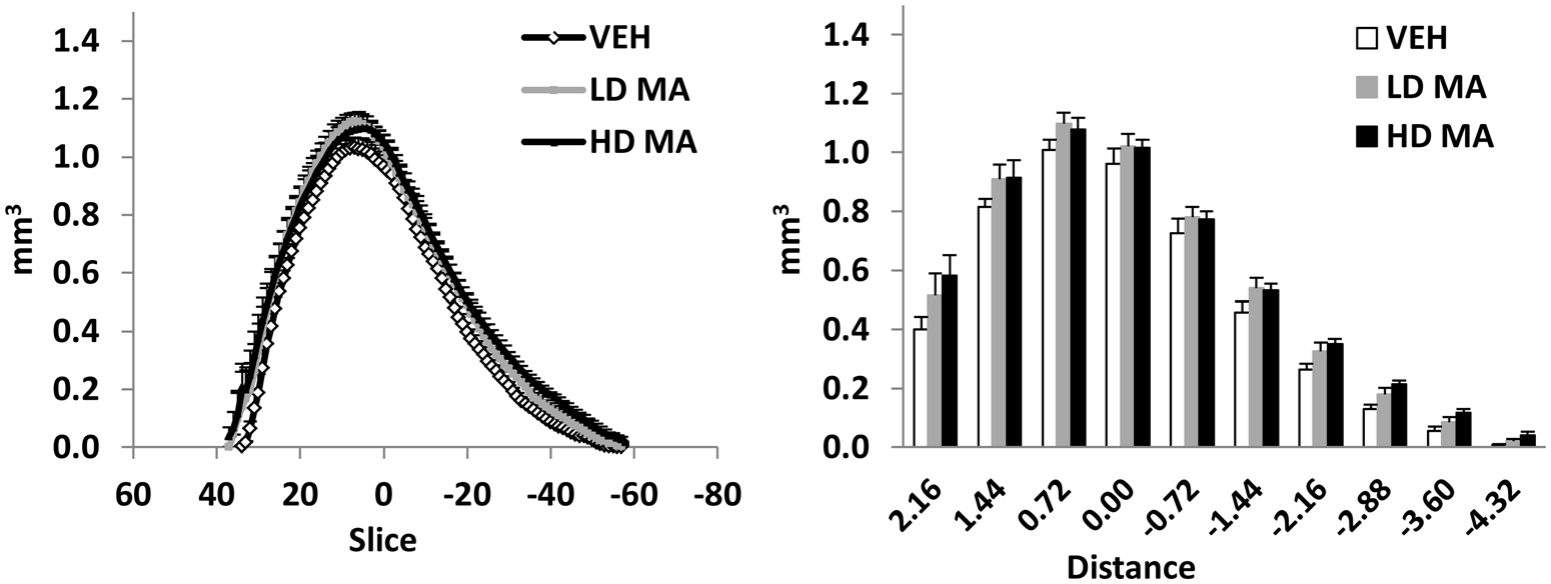
Volume of the striatum along the antroposterior axis. A Average volume of each MRI slice along the anteroposterior axis, from the three treatment groups. B Average volume of consecutive segments of the striatum, 720um each (9 slices), along the anteroposterior axis. The points and bars represent mean + S.E.M. Zero on the X axis corresponds to the posterior part of the anterior commissure. Distance is in mm.

The volumes of the regions of interest are presented in Fig 2 and Table 1. The volume of the striatum was significantly higher by 15% in rats treated with HD-MA, compared with the vehicle group (one-way ANOVA, *F* (2, 16) = 4.35, *p* = 0.031; Bonferroni post-hoc, *p* = 0.036). The volume increase was uniform throughout the striatum. No statistically significant treatment x slice interaction was observed for the volume of the striatum along the anteroposterior axis (Fig 3). No statistically significant differences in the volume of the total brain, the cerebral cortex, the hippocampus, the globus pallidus, the cerebellum, and the external capsule were observed between groups. Analysis of co-variance, taking into consideration total brain volume, did not yield further or different results.

**Table 1:** Effects of chronic methamphetamine administration on regional rat brain volume.

	Vehicle			4 mg/kg MA			8 mg/kg MA		
Striatum	87.2	±	3.6	99.6	±	2.7	**100.6**	**±**	**3.9** *
Globus pallidus	11.9	±	1.1	12.7	±	1.3	11.8	±	0.6
Cerebral cortex	748.7	±	9.5	803	±	11.4	763.4	±	17.2
Hippocampus	117.3	±	2.9	126.2	±	3.6	116.5	±	4.1
Cerebellum	324.3	±	7.9	362.1	±	17.9	348	±	9.5
External capsule	107.8	±	4.9	110	±	3.7	107.9	±	3.3
Total brain	2592.4	±	39	2708.7	±	95.2	2633.9	±	43.1

The numbers represent mean ± SEM, expressed in mm3.

* denotes significant difference from vehicle, p<0.05.

### [^3^H]PK 11195 Autoradiography

**Fig 4:**
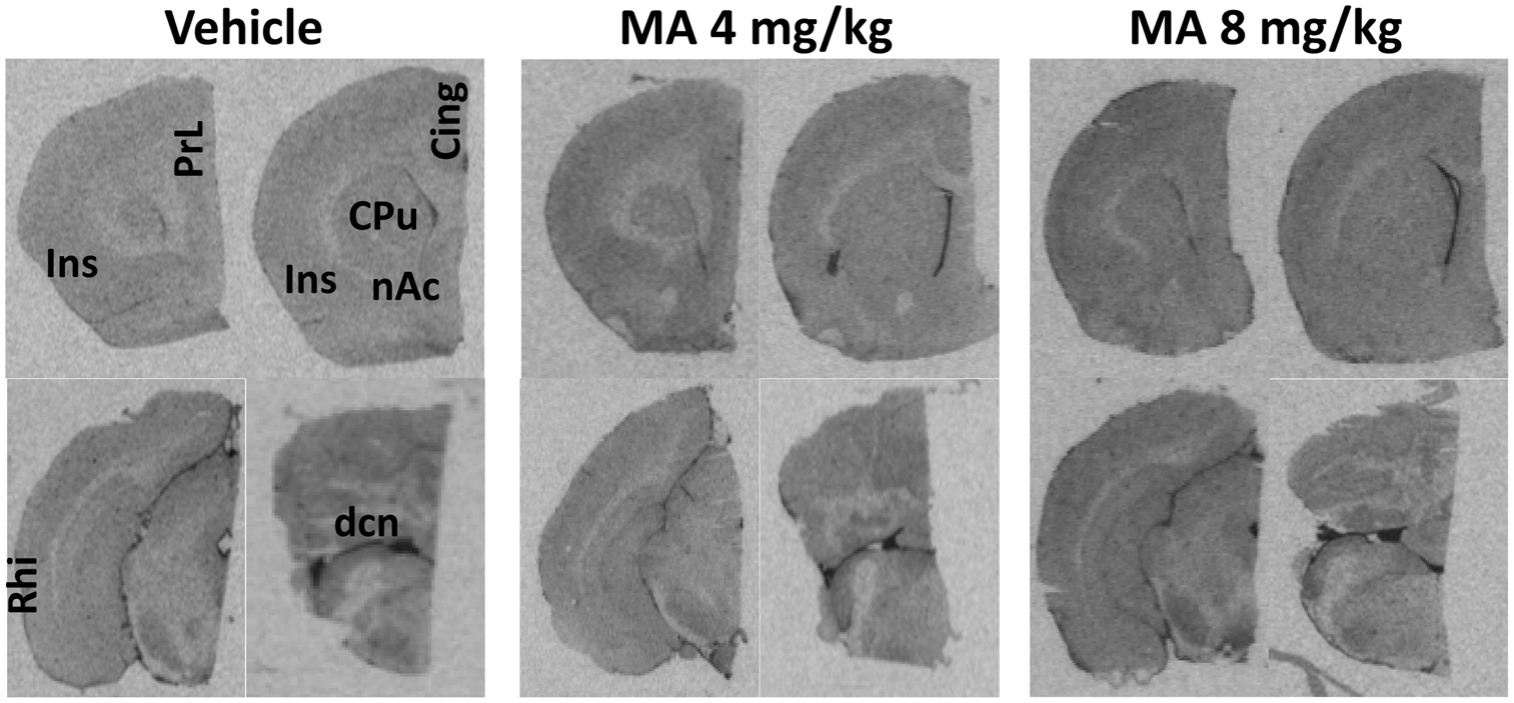
Chronic MA treatment increases [^3^H]PK 11195 binding in the brain. Representative in vitro autoradiographic images of [^3^H]PK 11195 binding in coronal brain sections of rats chronically treated with vehicle or with LD MA (4mg/kg) or with HD MA (8mg/kg). Significant effects of methamphetamine treatment on [^3^H]PK 11195 specific binding were observed in the highlighted areas, i.e. PrL: prelimbic cortex, Ins: insular cortex, Cing: cingulate cortex, CPu: caudate-putamen (striatum), nAc: nucleus accumbens, Rhi: rhinal cortex, dcn: deep cerebellar nuclei.

Representative sections showing the levels and distribution of [^3^H]PK 11195 binding in MA-treated and control rats are shown in Fig 4.

**Fig 5:**
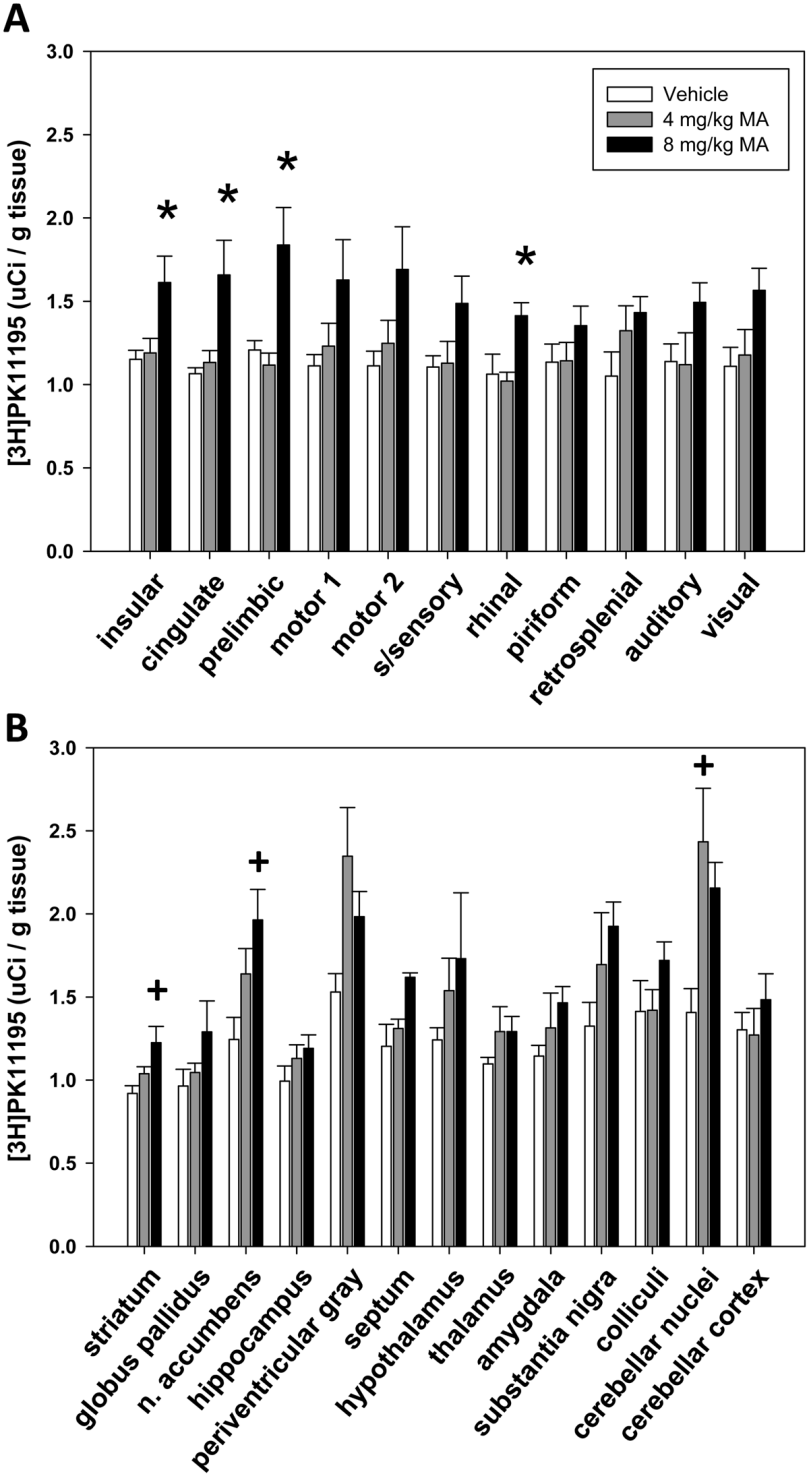
[3H]PK11195 specific binding in cerebral cortical (A) and subcortical (B) brain regions of rats chronically treated with vehicle or LD MA (4mg/kg) or HD MA (8 mg/kg). The bars represent mean + S.E.M. of [^3^H]PK11195 specific binding on fresh-frozen brain sections. * compared with vehicle and 4 mg/kg MA, + compared with vehicle.

Measures of [^3^H]PK 11195 specific binding were analyzed with one-way ANOVA. In cortical ROIs, a significant effect of treatment was found in the insular (*F* (2, 13) = 5.258, *p* < 0.05), cingulate (*F* (2, 13) = 6.490, *p* < 0.05), prelimbic (*F* (2, 13) = 7.817, *p* < 0.05), and rhinal (*F* (2, 13) = 4.993, *p* < 0.05) cortex. Pairwise comparisons using the Holm-Sidak method showed that in these regions HD MA treatment resulted in significantly higher specific [^3^H]PK 11195 binding over both vehicle and LD MA treatments (*p* <0.05; Fig 5a).

In subcortical ROIs, a significant effect of treatment was observed in the striatum (*F* (2, 13) = 5.172, *p* < 0.05), the nucleus accumbens (*F* (2, 13) = 4.861, *p* < 0.05), and the cerebellar nuclei (*F* (2, 13) = 6.186, *p* < 0.05). Pairwise comparisons showed that in the striatum (*p* = 0.008; Fig 5b) and the nucleus accumbens (*p* = 0.01; Fig 5b), HD MA significantly increased [^3^H]PK 11195 specific binding compared with vehicle. In the cerebellar nuclei, LD MA treatment significantly increased [^3^H]PK 11195, compared with vehicle (*p* = 0.008; Fig 5b).

### Brain Glucose Metabolism (BGluM)

**Fig 6:**
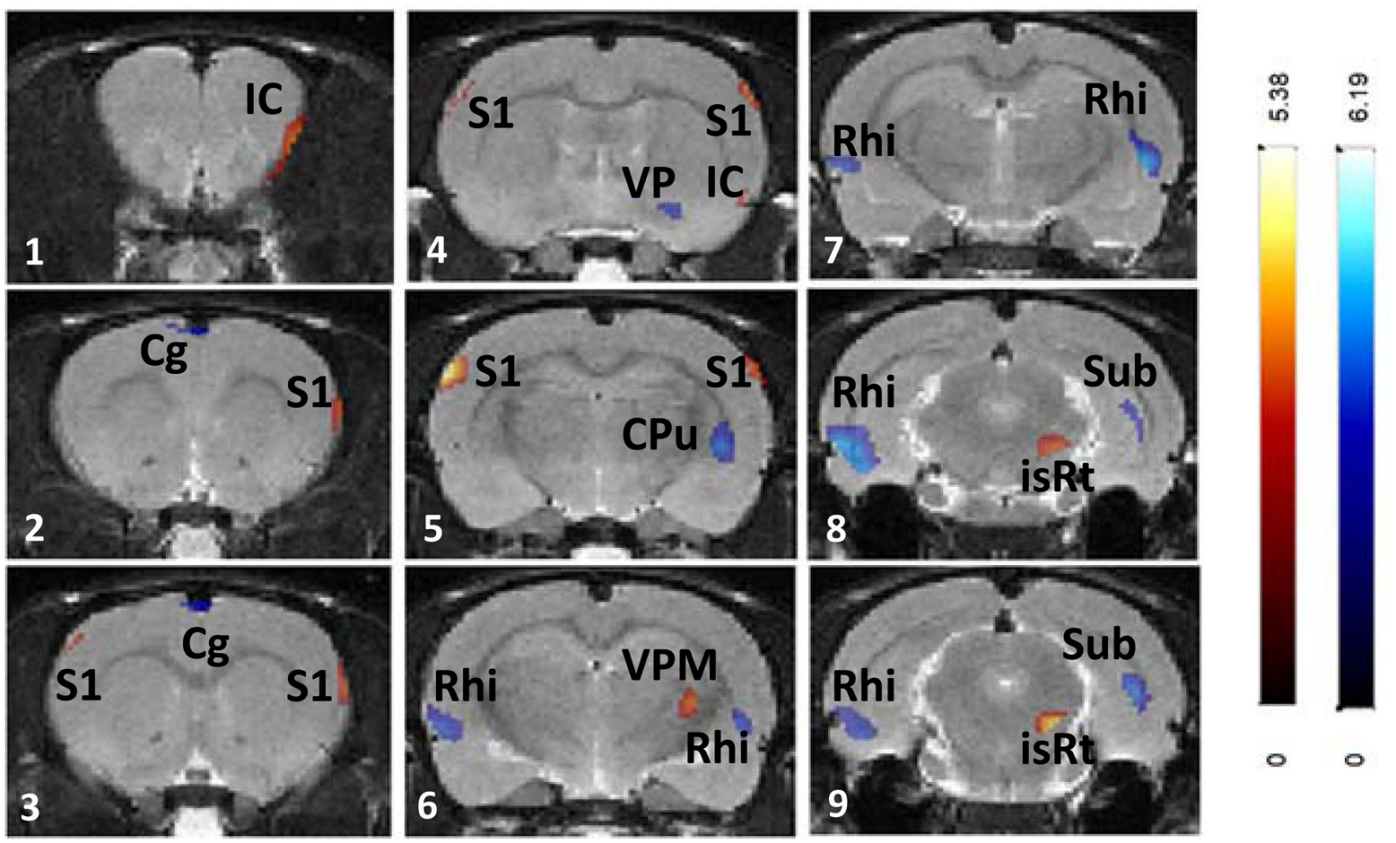
Effects of chronic LD MA (4mg/kg) treatment on regional brain glucose metabolism. Changes in regional brain glucose metabolism are superimposed on the Schweinhardt MRI template, in Paxinos space. Red: brain regions with increased glucose metabolism after 4 mg/kg MA treatment compared with vehicle treatment; Blue: brain regions with decreased glucose metabolism after 4mg/kg MA treatment compared with vehicle treatment. IC: insular cortex, Cg: cingulate cortex; S1: primary somatosensory cortex; VP: ventral pallidum; CPu: caudate-putamen (striatum); Rhi: rhinal cortices; VPM: ventral posteromedial thalamic nucleus; isRt: isthmic reticular formation; Sub: subiculum.

LD MA treatment resulted in increased BGluM (LD > Vehicle; Fig 6, Table 2) in insular cortex (panels 1–2), somatosensory cortex (panels 4–5), ventral posteromedial thalamic nucleus (panel 6), and isthmic reticular nucleus (panels 8–9). Decreased BGluM (LD < Vehicle; Table 2, Fig 6) was observed in cingulate cortex (panels 2–3), ventral pallidum (panel 4), tail of the striatum (panel 5), rhinal cortex (panels 6–9), and subiculum (panel 9).

**Table 2:** Brain regions showing significant differences in BGluM in rats treated with LD MA (4mg/kg) compared with vehicle.

*p* < 0.01 Ke > 50	Brain Region	Cluster Level				Stereotaxic Location of Peak (mm)		
		(Ke)	t value	z score	*p*	x	y	z
**Increased BGluM**	primary somatosensory cortex (S1)	328	5.38	4.06	0	6.3	3.2	-2.3
**LD MA > veh**	insular cortex (Ins)	177	3.76	3.16	0.001	-3.9	5	4.3
	ventral posteromedial thalamic nucleus (VPM)	145	3.43	2.95	0.002	-3.7	5.6	-3.3
**Decreased BGluM**	rhinal cortex (Rhi)	1039	6.19	4.42	0	6.7	6.8	-5.5
**LD MA < veh**	Striatum / subiculum (CPu/Sub)	773	4.63	3.68	0	-5.9	6.4	-4.3
	striatum-tail		4.15	3.4	0	-4.9	6.6	-2.7
	subiculum		4.07	3.36	0	-5.3	5.2	-7.1
	Cingulate cortex (Cg1)	108	3.84	3.21	0.001	1.0	1.0	1.3

LD: low dose; MA: methamphetamine; Veh: vehicle (saline); Increased BGluM: Significant clusters (Ke = 50) where metabolic activity was greater in LD MA treated rats compared with vehicle (α = .01); Decreased BGluM: Significant clusters (Ke = 50) where metabolic activity was lower in LD MA treated rats compared with vehicle (α = .01).

**Fig 7:**
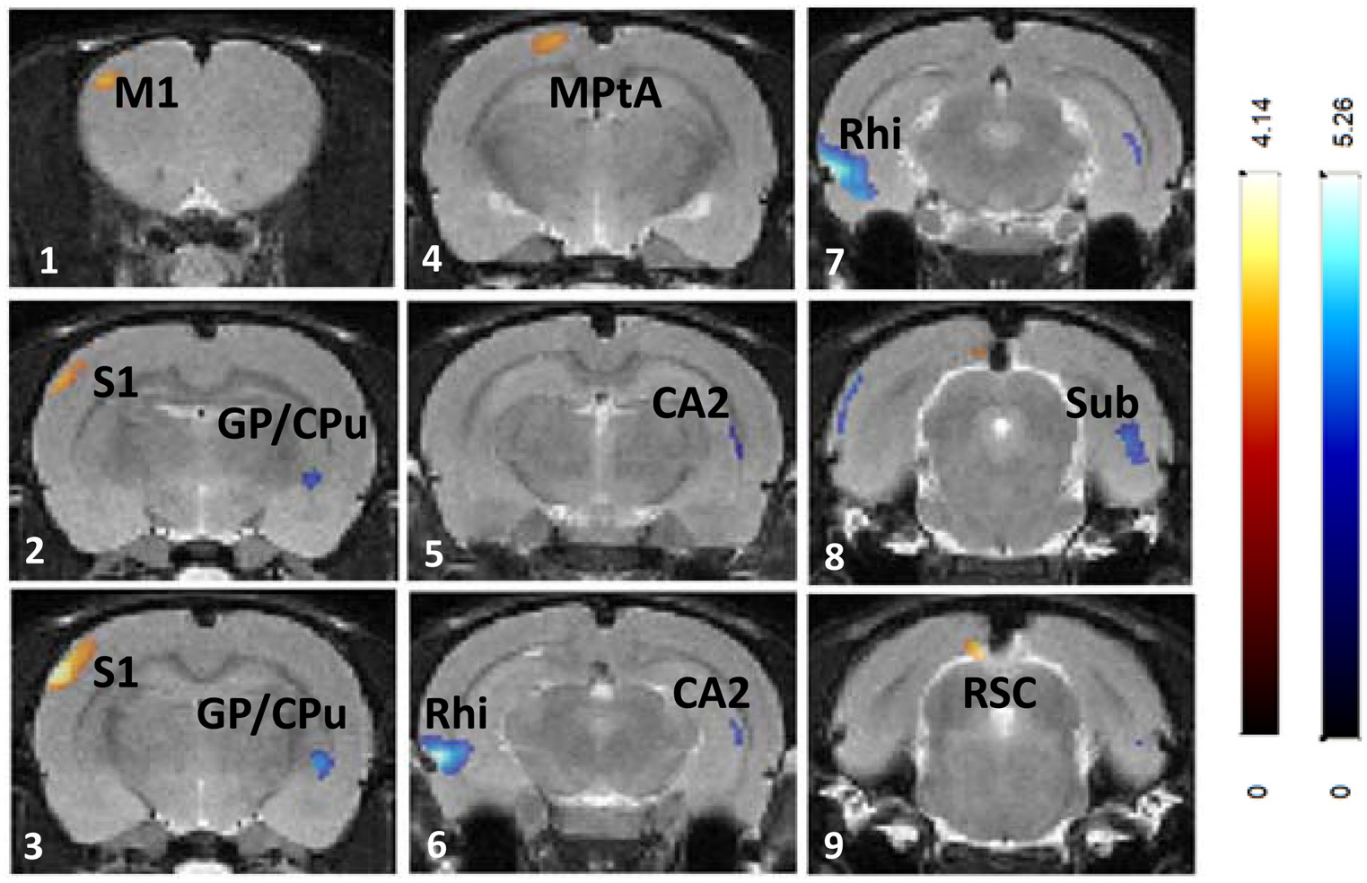
Effects of chronic HD MA (8mg/kg) treatment on regional brain glucose metabolism. Changes in regional brain glucose metabolism are superimposed on the Schweinhardt MRI template, in Paxinos space. Red: brain regions with increased glucose metabolism after 8mg/kg MA treatment compared with vehicle treatment; Blue: brain regions with decreased glucose metabolism after MA 8mg/kg treatment compared with vehicle treatment. M1: primary motor cortex; S1: primary somatosensory cortex; CPu: caudate-putamen (striatum); GP: globus pallidus; PtA: parietal association area; CA1: cornu ammonis 2 (hippocampus); Rhi: rhinal cortices; Sub: subiculum; RCS: retrosplenial cortex.

HD MA treatment resulted in increased BGluM (HD > Vehicle; Fig 7, Table 3) in primary somatosensory cortex (panels 1–3), parietal association area (panel 4), and retrosplenial cortex (panel 9). Decreased BGluM (HD < Vehicle; Table 3, Fig 7) was observed in globus pallidus and tail of the striatum (panel 3), hippocampus (CA2 region, panels 5–6), and rhinal cortex (panels 6–7).

**Table 3:** Brain regions showing significant differences in BGluM in rats treated with HD MA (8mg/kg) compared with vehicle.

*p* < 0.01 Ke > 50	Brain Region	Cluster Level				Stereotaxic Location of Peak (mm)		
		(Ke)	t value	z score	*p*	x	y	z
**Increased BGluM**	primary somatosensory cortex (S1)	251	4.14	3.39	0	6.1	3	-2.3
**HD MA > Veh**	retrosplenial cortex (RSC)	73	3.47	2.98	0.001	1.1	1.8	-8.5
	parietal association area (MPtA)	91	2.99	2.61	0.004	1.9	8	-3.3
**Decreased BGluM**	rhinal cortex (Rhi)	694	5.26	4	0	6.7	6.6	-5.9
**HD MA < Veh**	Subiculum / Hippocampus(Sub/Hipp)	428	3.97	3.29	0	-5.3	5.2	-7.3
	hippocampus		3.39	2.92	0.002	-5.5	5.6	-5.1
	globus pallidus / striatum tail (GP/CPu)	92	3.72	3.14	0.001	-4.7	6.8	-2.5

HD: high dose (8mg/kg); MA methamphetamine; Veh: vehicle (saline); Increased BGluM: Significant clusters (Ke = 50) where metabolic activity was greater in HD MA treated rats compared with vehicle (α = .01); Decreased BGluM: Significant clusters (Ke = 50) where metabolic activity was lower in HD MA treated rats compared with vehicle (α = .01).

**Fig 8:**
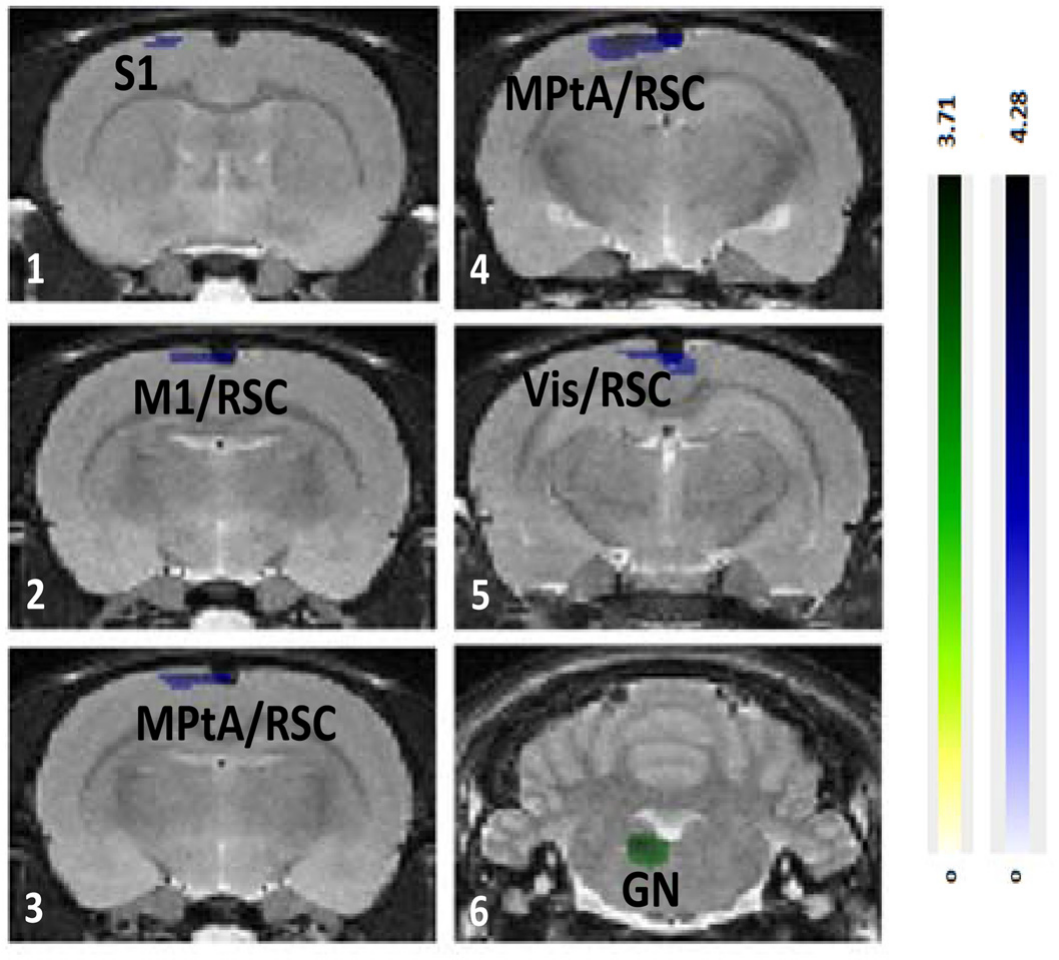
Differential effects of 4 and 8 mg/kg MA treatments on regional brain glucose metabolism. Regional brain glucose metabolism differences between 4 and 8 mg/kg MA treatments, superimposed on the Schweinhardt MRI template, in Paxinos space. Green: brain regions with increased glucose metabolism after 4mg/kg MA treatment compared with 8 mg/kg MA treatment. Blue: brain regions with decreased glucose metabolism after 4mg/kg MA treatment compared with 8 mg/kg MA treatment. S1: primary somatosensory cortex; M1: primary motor cortex; RSC: retrosplenial cortex; PtA: parietal association area; Vis: visual cortex; RSC: retrosplenial cortex; GN: gigantocellular nucleus.

Between MA doses, increased BGluM with HD MA (HD MA > LD MA; Fig 8, Table 4) was observed in parietal association area (panels 3–4), retrosplenial cortex (panels 3–5), and somatosensory cortex (panel 1). Increased BGluM with LD MA (LD MA > HD MA; Fig 8, Table 4) was observed in the temporal association area and paragigantocellular nucleus (panel 6).

**Table 4:** Brain regions showing significant differences in BGluM in rats treated with LD MA (4mg/kg) compared with HD MA (8 mg/kg).

*p* < 0.01 Ke > 50	Brain Region	Cluster Level				Stereotaxic Location of Peak (mm)		
		(Ke)	t value	z score	*p*	x	y	z
**LD MA> HD MA**	temporal association area (TeA)	65	3.71	3.13	0.001	-7.3	4.2	-7.1
	Paragigantocellular Nucleus (Gn)	164	3.55	3.03	0.001	1.1	7.6	-11.1
**LD MA< HD MA**	parietal association / primary somatosensory / retrosplenial cortex	822	4.28	3.48	0	1.7	0.6	-3.3
	primary somatosensory cortex (S1)		3.1	2.72	0.003	2.3	0.8	-0.7
	retrosplenial cortex (RSC)		2.87	2.55	0.005	-0.5	0.6	-4.1

LD: low dose (4mg/kg); HD: high dose (8 mg/kg); MA: methamphetamine; LD MA > HD mg/kg MA: Significant clusters (Ke = 50) where metabolic activity was greater in LD MA treated rats compared to HD MA treated rats (α = .01); LD MA < HD mg/kg MA: Significant clusters (Ke = 50) where metabolic activity was lower in LD MA treated rats compared with HD MA treated rats (α = .01).

## Discussion

Here we show that a daily dose of HD MA (8 mg/kg) for four months can cause increases in the volume of the striatum and increases in microglial activation in the striatum. Chronic MA treatment also resulted in significant changes in BGluM. Increases in BGluM were found in several areas including primary and higher order somatosensory regions, while decreases in BGluM were found in the tail of the striatum, hippocampus, and rhinal cortices.

### HD MA-treated rats present enlarged striatum

In the current study, enlarged striatal volume was observed after chronic MA treatment. A similar finding was also observed in clinical and non-human primate studies that report increases in striatal volume in abstinent as well as active MA users [12–17]. Although structural brain deficits may pre-date MA use in humans, the coincidence among rat, monkey, and human findings allows us to suggest that enlarged striatal volume is a major and long-lasting effect of chronic MA use.

In addition, a unique aspect of this study is that the onset of MA treatment began at adolescence. Human studies on the effects of chronic MA use exclusively in adolescence [15] or chronic MA use starting at 12–34 years and extending into adulthood [16] report an increase in the volume of the striatum, which is in agreement with our current anatomical findings and confers clinical relevance to our rodent model of chronic MA exposure.

### HD MA-treated rats present microglial activation in the striatum

It is well established that the peripheral benzodiazepine binding site, as determined with in vitro and in vivo PK 11195 binding, is localized in activated microglia [34–37]. Activated microglia may be cytotoxic, through the release of free oxygen species, nitrogen oxides such as NO, and pro-inflammatory cytokines such as IFN-γ [38, 39]. In addition, microglial activation takes place after neurotoxic amphetamine treatment [40] and before dopamine terminal pathology [41], and could thus account for the impairment of dopamine neuron integrity and dopamine system function observed after chronic methamphetamine use (Thanos et al in preparation, [42]).

On the other hand, activated microglia also have a protective role for the brain, since they promote neurogenesis [43], they remove toxic glutamate levels [44], and they contribute to tissue remodeling be removing cellular debris [38]. Most importantly, they express and release a number of neurotrophic factors, including nerve growth factor (NGF), brain derived neurotrophic factor (BDNF), glial cell line-derived neurotrophic factor (GDNF), fibroblast growth factor (FGF) [38, 39], and they induce dopamine terminal sprouting through the release of BDNF and GDNF in the striatum [45, 46].

Given that BNDF enrichment of the brain can produce volumetric increases [47], we could hypothesize the existence of a pathway linking methamphetamine exposure to microglia activation, neurotrophin secretion, and increased volume in the striatum. Such a mechanism could explain the positive association between striatal volume and measures of cognitive function observed in human methamphetamine users [13, 16]. However, the present study indicates only an association between microglial activation and changes in brain volume. Further research is warranted to prove that a causal link between increased microglial activation and changes in brain volume indeed exists after chronic MA treatment.

### MA treatment causes significant microglial activation in DA-poor brain regions

Although much of the focus on MA effects on the brain has been in the striatum, here we extend our findings from the striatum and show microglial activation in other areas of the brain. LD MA treatment resulted in increased [^3^H]PK 11195 specific binding in the cerebellar nuclei compared with vehicle. HD MA treatment increased [^3^H]PK 11195 binding in the insular, cingulate, prelimbic, and rhinal cortices, in addition to increases in the striatum and the nucleus accumbens. However, these cortical and cerebellar regions with increased [^3^H]PK 11195 binding did not show a significant change in their volume. This could suggest that increased [^3^H]PK 11195 binding does not always correlate to an increase in volume in all brain regions. It is possible that in these areas, with weak DA innervation, microglial activation may follow a different timeline in correlating with brain morphology and/or a different line of function. In addition to its effects on dopaminergic cells, MA also affects neuronal function in cells outside of the dopaminergic system [6, 48]. Indeed, a loss of serotonin and its uptake sites, loss of norepinephrine, and changes in GABA and glutamate transmission have all been reported [6]. Hence, subsequent microglial responses in these areas of the brain, that are known to be affected by MA [7], were expected.

These preclinical findings have been extended to abstinent human MA users, showing increased [^11^C]PK 11195 binding in many distinct brain regions in addition to the striatum, including midbrain, thalamus, insular cortex, and orbitofrontal cortex [26]. In addition to increased PK 11195 binding, other markers for inflammation, including widespread increases in proinflammatory cytokines and chemokines have been observed in both animals and humans [49, 50]. These results show that MA-induced effects can extend beyond dopaminergic cells in the striatum, to cells outside the dopaminergic pathways. Finally, MA abuse is associated with significant reduction in cerebral blood flow which could render neuronal tissue susceptible to damage from hypoxia which would also contribute to gliosis [51, 52]. These findings all suggest that MA may induce global microglial activation and subsequent widespread changes within the brain [21, 26]. However, the exact cause of MA-induced increases in PK 11195 binding has yet to be identified and the extent to which these findings translate to a behavioral phenotype remain unclear.

### Increased BGluM after chronic MA treatment

LD MA treatment resulted in increased BGluM in the ventral posteromedial thalamic nucleus, in face somatosensory cortex (whisker and upper lip), in the reticular formation, and in the insular cortex. The ventral posteromedial thalamic nucleus projects to the somatosensory cortex [53], and in particular to the whisker somatosensory cortex, in which increased BGluM was also observed in LD MA-treated rats. These effects predict changes in face sensory processing after extended MA exposure. Indeed, exposure to MA has been previously shown to induce damage to whisker somatosensory cortical neurons, which was associated with increased stereotypical whisker movements [54] and was prevented by the removal of whiskers [55, 56].

In addition, increased BGluM was also observed in the insular cortex and reticular formation. The insular cortex receives multi-modal sensory input from thalamic nuclei and forms reciprocal connections with the limbic system [57, 58]. It has been proposed that the insular cortex is involved in a variety of functions, including salience detection [59, 60]. Similarly, the reticular formation is involved in sleep cycles, more specifically in promoting wakefulness and inhibiting REM sleep [61, 62]. Increased BGluM in the insular cortex and reticular formation during LD MA exposure are especially significant in light of past findings where MA has been shown to be effective in treating narcolepsy and inducing increased alertness in humans [63, 64].

HD MA treatment resulted in increased BGluM in the whisker somatosensory cortex, the parietal association area, and the retrosplenial cortex. Similar to LD MA treatment, increased BGluM was specific to the barrel field region of the somatosensory cortex, indicating effects of HD MA on face sensory processing. The higher BGluM in the HD MA the parietal area is in agreement with the increased BGluM in the parietal cortex of MA users previously reported in clinical studies [18, 20]. These patients showed impairment in the Grooved Pegboard task, in which fine motor coordination is assessed, indicating a functional link with hyperactivity in the parietal cortex [22]. More recently, fMRI studies in MA users have shown activation of parietal regions during a delayed discounting task, where MA users were more likely to select a small immediate reward [65].

In rodents, in addition to thalamocortical projections, sensory areas within the neocortex are also known to project to the parietal association areas [66]. Im mammals, these regions are involved in exploratory behavior and spatial recognition by use of the digits, hands, and limbs, and in rodents the whiskers and snout [66]. Furthermore, the parietal association areas and the retrosplenial cortex are two of the regions thought to be involved in the rodent default mode network (DMN) [67]. The DMN is a set of interconnected brain regions, found to be less active when an individual is focused and engaged in a task [68, 69]. Lu and colleagues [67] proposed that the DMN in rats serves to integrate sensory information to guide behavior in anticipation to changing environmental stimuli. Overall, the increased BGluM in the parietal association area and the retrosplenial cortex are indicative of HD MA effects on higher order sensorimotor processing and cognitive control.

### Decreased BGluM after Chronic MA Treatment

Regions that showed decreased BGluM during exposure to LD MA include interconnected areas of the limbic system, including cingulate cortex, rhinal cortices, and subiculum. The cingulate cortex sends projections to the rhinal cortices, which, in turn, projects onto the subiculum [58]. These connections are reciprocal, as the subiculum projects back to the rhinal cortices, which in turn project back to the cingulate cortex [58]. Similar to LD MA, HD MA treatment decreased BGluM in limbic areas (rhinal cortex, subiculum). This effect was extended in HD MA treated rats and a decrease in BGluM in the hippocampus, a region strongly connected with both the subiculum and rhinal cortices, was also observed, in agreement with previous in vitro 2- Deoxy-D-glucose (2DG) studies [70]. Overall, the results predict that long-term MA treatment may affect brain function related to memory and navigation.

Two regions of the basal ganglia, the tail of the striatum and the globus pallidus, showed decreased BGluM after chronic MA treatment, in agreement with previous observations in human MA users [18] and with rat 2DG studies [70]. Decreased BGluM in these two basal ganglia areas may indicate MA effects on parts of the limbic system as well as the basal ganglia motor loop. The tail of the striatum is part of the limbic basal ganglia with known anatomical connections with the amygdala [71]. Since the striatum is a DA-rich region of the brain, effects of MA treatment in this area can be attributed to the direct effects of the drug. On the other hand, the globus pallidus is a region with little dopaminergic innervation and with strong striatal inputs. Thus, decreased metabolic activity in this region may reflect an indirect consequence of MA-induced changes on DA cells and the nigro-striato-pallidal projections, as previously suggested [72].

The increased metabolic activity in the brain of MA users has been speculated to reflect neuroinflammation and gliosis [18]. In this study, the only brain region that showed increased BGluM, in conjunction with increased [^3^H]PK 11195 binding, was the insular cortex of LD MA treated rats. However, regions that showed decreased BGluM also showed increases in [^3^H]PK 11195 (rhinal cortex, cingulate cortex, striatum), suggesting that increased metabolic activity is not necessarily a consequence of neuroinflammation.

### Limitations of the study

The three experiments of the current study, MRI volumetrics, activated microglia assessment, and BGluM assessment were performed in different groups of animals, which does not allow correlation analyses that would shed more light into the associations between the three measures, without, however, providing proof of causality. The need for future studies, including a study on how the time course of microglial activation corresponds to an increase in striatal volume and/or a stereological volume estimation in conjunction with microglial assessment in the same tissue, and are deemed necessary.

MA was dissolved in saline, without further pH adjustments, according to previously published protocols. Since the pH of aqueous MA solution is 6 [73] peripheral inflammatory effects that would induce brain microglia activation [74] cannot be excluded, particularly since MA was administered for a long period of time. On the other hand, human users also consume MA hydrochloride, and the aqueous solutions of crystal meth are reported to be acidic, the majority below 5.5 [75]. In addition, MA users take MA on a daily basis and for many years (e.g. see [13]), a condition that we wished to reproduce in the current study. Taking all the above under consideration and including the fact that the rats did not have visible distress signs, visible signs of overall hyperalgesia or swelling and pain at or near the injection sites, we may conclude that—in spite of its limitation regarding the pH—the animal model we chose reproduces several aspects of human MA abuse and retains its clinical relevance. In future experiments, MA should be dissolved in PBS or the pH of the NaCl solution should be adjusted to 7, with dilute NaOH.

### Summary

Overall, the current study shows an increase in striatal volume after prolonged treatment with 8 mg/kg/day MA, along with increased microglial activation in the striatum and decreased BGluM in the caudal striatum. The increased microglial response extends beyond the striatum, to distinct dopamine-poor brain regions, not showing volumetric changes. BGluM changes in DA-related regions (globus pallidus, striatum) may be the result of a response to MA by the dopaminergic system. In contrast, the changes in BGluM in other areas (cerebral cortex, hippocampus) suggest MA effects on other neurotransmitter pathways. BGluM changes in dopamine-poor regions include increases in primary and higher-order sensory cortical areas, insular and retrosplenial cortices, and the reticular formation, while decreases are observed in the rhinal cortices, hippocampus, and subiculum.

## References

[1] Winslow BT, Voorhees KI, Pehl KA. Methamphetamine abuse. American family physician. 2007;76(8):1169–74. . 17990840

[2] Sulzer D, Sonders MS, Poulsen NW, Galli A. Mechanisms of neurotransmitter release by amphetamines: a review. Progress in neurobiology. 2005;75(6):406–33. 10.1016/j.pneurobio.2005.04.003 . 15955613

[3] Thrash B, Thiruchelvan K, Ahuja M, Suppiramaniam V, Dhanasekaran M. Methamphetamine-induced neurotoxicity: the road to Parkinson's disease. Pharmacological reports: PR. 2009;61(6):966–77. . 20081231 10.1016/s1734-1140(09)70158-6

[4] Ferrucci M, Giorgi FS, Bartalucci A, Busceti CL, Fornai F. The effects of locus coeruleus and norepinephrine in methamphetamine toxicity. Current neuropharmacology. 2013;11(1):80–94. 10.2174/157015913804999522 23814540 PMC3580794

[5] Halpin LE, Collins SA, Yamamoto BK. Neurotoxicity of methamphetamine and 3,4-methylenedioxymethamphetamine. Life sciences. 2014;97(1):37–44. 10.1016/j.lfs.2013.07.014 23892199 PMC3870191

[6] Krasnova IN, Cadet JL. Methamphetamine toxicity and messengers of death. Brain research reviews. 2009;60(2):379–407. 10.1016/j.brainresrev.2009.03.002 19328213 PMC2731235

[7] Gold MS, Kobeissy FH, Wang KK, Merlo LJ, Bruijnzeel AW, Krasnova IN, et al Methamphetamine- and trauma-induced brain injuries: comparative cellular and molecular neurobiological substrates. Biological psychiatry. 2009;66(2):118–27. 10.1016/j.biopsych.2009.02.021 19345341 PMC2810951

[8] Volkow ND, Fowler JS, Wang GJ, Shumay E, Telang F, Thanos PK, et al Distribution and pharmacokinetics of methamphetamine in the human body: clinical implications. PloS one. 2010;5(12):e15269 10.1371/journal.pone.0015269 21151866 PMC2998419

[9] Chin KM, Channick RN, Rubin LJ. Is methamphetamine use associated with idiopathic pulmonary arterial hypertension? Chest. 2006;130(6):1657–63. 10.1378/chest.130.6.1657 . 17166979

[10] Ishigami A, Tokunaga I, Gotohda T, Kubo S. Immunohistochemical study of myoglobin and oxidative injury-related markers in the kidney of methamphetamine abusers. Legal medicine. 2003;5(1):42–8. . 12935649 10.1016/s1344-6223(03)00005-1

[11] Thompson PM, Hayashi KM, Simon SL, Geaga JA, Hong MS, Sui Y, et al Structural abnormalities in the brains of human subjects who use methamphetamine. The Journal of neuroscience: the official journal of the Society for Neuroscience. 2004;24(26):6028–36. 10.1523/JNEUROSCI.0713-04.2004 . 15229250 PMC6729247

[12] Chang L, Alicata D, Ernst T, Volkow N. Structural and metabolic brain changes in the striatum associated with methamphetamine abuse. Addiction. 2007;102 Suppl 1:16–32. 10.1111/j.1360-0443.2006.01782.x . 17493050

[13] Chang L, Cloak C, Patterson K, Grob C, Miller EN, Ernst T. Enlarged striatum in abstinent methamphetamine abusers: a possible compensatory response. Biological psychiatry. 2005;57(9):967–74. 10.1016/j.biopsych.2005.01.039 . 15860336 PMC4899039

[14] Jernigan TL, Gamst AC, Archibald SL, Fennema-Notestine C, Mindt MR, Marcotte TD, et al Effects of methamphetamine dependence and HIV infection on cerebral morphology. The American journal of psychiatry. 2005;162(8):1461–72. 10.1176/appi.ajp.162.8.1461 . 16055767

[15] Churchwell JC, Carey PD, Ferrett HL, Stein DJ, Yurgelun-Todd DA. Abnormal striatal circuitry and intensified novelty seeking among adolescents who abuse methamphetamine and cannabis. Developmental neuroscience. 2012;34(4):310–7. 22986770 10.1159/000337724 PMC3513364

[16] Jan RK, Lin JC, Miles SW, Kydd RR, Russell BR. Striatal volume increases in active methamphetamine-dependent individuals and correlation with cognitive performance. Brain sciences. 2012;2(4):553–72. 10.3390/brainsci2040553 24961260 PMC4061811

[17] Groman SM, Morales AM, Lee B, London ED, Jentsch JD. Methamphetamine-induced increases in putamen gray matter associate with inhibitory control. Psychopharmacology. 2013;229(3):527–38. 10.1007/s00213-013-3159-9 23748383 PMC3770792

[18] Volkow ND, Chang L, Wang GJ, Fowler JS, Franceschi D, Sedler MJ, et al Higher cortical and lower subcortical metabolism in detoxified methamphetamine abusers. The American journal of psychiatry. 2001;158(3):383–9. 10.1176/appi.ajp.158.3.383 . 11229978

[19] Wang GJ, Volkow ND, Chang L, Miller E, Sedler M, Hitzemann R, et al Partial recovery of brain metabolism in methamphetamine abusers after protracted abstinence. The American journal of psychiatry. 2004;161(2):242–8. 10.1176/appi.ajp.161.2.242 . 14754772

[20] Berman SM, Voytek B, Mandelkern MA, Hassid BD, Isaacson A, Monterosso J, et al Changes in cerebral glucose metabolism during early abstinence from chronic methamphetamine abuse. Molecular psychiatry. 2008;13(9):897–908. 10.1038/sj.mp.4002107 17938635 PMC2786221

[21] London ED, Simon SL, Berman SM, Mandelkern MA, Lichtman AM, Bramen J, et al Mood disturbances and regional cerebral metabolic abnormalities in recently abstinent methamphetamine abusers. Archives of general psychiatry. 2004;61(1):73–84. 10.1001/archpsyc.61.1.73 . 14706946

[22] Volkow ND, Chang L, Wang GJ, Fowler JS, Leonido-Yee M, Franceschi D, et al Association of dopamine transporter reduction with psychomotor impairment in methamphetamine abusers. The American journal of psychiatry. 2001;158(3):377–82. 10.1176/appi.ajp.158.3.377 . 11229977

[23] Volkow ND, Chang L, Wang GJ, Fowler JS, Ding YS, Sedler M, et al Low level of brain dopamine D2 receptors in methamphetamine abusers: association with metabolism in the orbitofrontal cortex. The American journal of psychiatry. 2001;158(12):2015–21. 10.1176/appi.ajp.158.12.2015 . 11729018

[24] Volkow ND, Chang L, Wang GJ, Fowler JS, Franceschi D, Sedler M, et al Loss of dopamine transporters in methamphetamine abusers recovers with protracted abstinence. The Journal of neuroscience: the official journal of the Society for Neuroscience. 2001;21(23):9414–8. . 11717374 10.1523/JNEUROSCI.21-23-09414.2001 PMC6763886

[25] Volkow ND, Wang GJ, Smith L, Fowler JS, Telang F, Logan J, et al Recovery of dopamine transporters with methamphetamine detoxification is not linked to changes in dopamine release. NeuroImage. 2015;121:20–8. 10.1016/j.neuroimage.2015.07.035 . 26208874

[26] Sekine Y, Ouchi Y, Sugihara G, Takei N, Yoshikawa E, Nakamura K, et al Methamphetamine causes microglial activation in the brains of human abusers. The Journal of neuroscience: the official journal of the Society for Neuroscience. 2008;28(22):5756–61. 10.1523/JNEUROSCI.1179-08.2008 18509037 PMC2491906

[27] Thanos PK, Robison L, Nestler EJ, Kim R, Michaelides M, Lobo MK, et al Mapping brain metabolic connectivity in awake rats with muPET and optogenetic stimulation. The Journal of neuroscience: the official journal of the Society for Neuroscience. 2013;33(15):6343–9. 10.1523/JNEUROSCI.4997-12.2013 23575833 PMC3666931

[28] Studholme C, Hill DLG, Hawkes DJ. An overlap invariant entropy measure of 3D medical image alignment. Pattern Recogn. 1999;32(1):71–86. 10.1016/S0031-3203(98)00091-0. WOS:000078298000006.

[29] Paxinos G, Watson C. The rat brain in stereotaxic coordinates. 6th ed: Elsevier Academic Press; 2007.

[30] Liraz-Zaltsman S, Alexandrovich AG, Trembovler V, Fishbein I, Yaka R, Shohami E, et al Regional sensitivity to neuroinflammation: in vivo and in vitro studies. Synapse. 2011;65(7):634–42. 10.1002/syn.20889 21108236 PMC3075319

[31] Dhawan J, Benveniste H, Nawrocky M, Smith SD, Biegon A. Transient focal ischemia results in persistent and widespread neuroinflammation and loss of glutamate NMDA receptors. NeuroImage. 2010;51(2):599–605. 10.1016/j.neuroimage.2010.02.073 20206701 PMC2856923

[32] Michaelides M, Pascau J, Gispert JD, Delis F, Grandy DK, Wang GJ, et al Dopamine D4 receptors modulate brain metabolic activity in the prefrontal cortex and cerebellum at rest and in response to methylphenidate. The European journal of neuroscience. 2010;32(4):668–76. 10.1111/j.1460-9568.2010.07319.x 20646063 PMC2938021

[33] Schweinhardt P, Fransson P, Olson L, Spenger C, Andersson JL. A template for spatial normalisation of MR images of the rat brain. Journal of neuroscience methods. 2003;129(2):105–13. . 14511814 10.1016/s0165-0270(03)00192-4

[34] Myers R, Manjil LG, Cullen BM, Price GW, Frackowiak RS, Cremer JE. Macrophage and astrocyte populations in relation to [3H]PK 11195 binding in rat cerebral cortex following a local ischaemic lesion. Journal of cerebral blood flow and metabolism: official journal of the International Society of Cerebral Blood Flow and Metabolism. 1991;11(2):314–22. 10.1038/jcbfm.1991.64 . 1997503

[35] Stephenson DT, Schober DA, Smalstig EB, Mincy RE, Gehlert DR, Clemens JA. Peripheral benzodiazepine receptors are colocalized with activated microglia following transient global forebrain ischemia in the rat. The Journal of neuroscience: the official journal of the Society for Neuroscience. 1995;15(7 Pt 2):5263–74. . 7623150 10.1523/JNEUROSCI.15-07-05263.1995 PMC6577872

[36] Vowinckel E, Reutens D, Becher B, Verge G, Evans A, Owens T, et al PK11195 binding to the peripheral benzodiazepine receptor as a marker of microglia activation in multiple sclerosis and experimental autoimmune encephalomyelitis. Journal of neuroscience research. 1997;50(2):345–53. . 9373043 10.1002/(SICI)1097-4547(19971015)50:2<345::AID-JNR22>3.0.CO;2-5

[37] Raghavendra Rao VL, Dogan A, Bowen KK, Dempsey RJ. Traumatic brain injury leads to increased expression of peripheral-type benzodiazepine receptors, neuronal death, and activation of astrocytes and microglia in rat thalamus. Experimental neurology. 2000;161(1):102–14. 10.1006/exnr.1999.7269 . 10683277

[38] Kreutzberg GW. Microglia: a sensor for pathological events in the CNS. Trends in neurosciences. 1996;19(8):312–8. . 8843599 10.1016/0166-2236(96)10049-7

[39] Nakajima K, Kohsaka S. Microglia: activation and their significance in the central nervous system. Journal of biochemistry. 2001;130(2):169–75. . 11481032 10.1093/oxfordjournals.jbchem.a002969

[40] Thomas DM, Walker PD, Benjamins JA, Geddes TJ, Kuhn DM. Methamphetamine neurotoxicity in dopamine nerve endings of the striatum is associated with microglial activation. The Journal of pharmacology and experimental therapeutics. 2004;311(1):1–7. 10.1124/jpet.104.070961 . 15163680

[41] LaVoie MJ, Card JP, Hastings TG. Microglial activation precedes dopamine terminal pathology in methamphetamine-induced neurotoxicity. Experimental neurology. 2004;187(1):47–57. 10.1016/j.expneurol.2004.01.010 . 15081587

[42] Kim R, Rocco MJ, Delis F, Wang GJ, Volkow ND, Thanos PK, editor Methamphetamine Effects on the Dopamine Transporter (DAT), D1 (D1R) and D2 Receptor (D2R) in Rats. Society for Neuroscience; 2011 11 2011; Washington D.C.

[43] Butovsky O, Ziv Y, Schwartz A, Landa G, Talpalar AE, Pluchino S, et al Microglia activated by IL-4 or IFN-gamma differentially induce neurogenesis and oligodendrogenesis from adult stem/progenitor cells. Molecular and cellular neurosciences. 2006;31(1):149–60. 10.1016/j.mcn.2005.10.006 . 16297637

[44] Persson M, Brantefjord M, Hansson E, Ronnback L. Lipopolysaccharide increases microglial GLT-1 expression and glutamate uptake capacity in vitro by a mechanism dependent on TNF-alpha. Glia. 2005;51(2):111–20. 10.1002/glia.20191 . 15789431

[45] Batchelor PE, Liberatore GT, Wong JY, Porritt MJ, Frerichs F, Donnan GA, et al Activated macrophages and microglia induce dopaminergic sprouting in the injured striatum and express brain-derived neurotrophic factor and glial cell line-derived neurotrophic factor. The Journal of neuroscience: the official journal of the Society for Neuroscience. 1999;19(5):1708–16. . 10024357 10.1523/JNEUROSCI.19-05-01708.1999 PMC6782182

[46] Batchelor PE, Porritt MJ, Martinello P, Parish CL, Liberatore GT, Donnan GA, et al Macrophages and Microglia Produce Local Trophic Gradients That Stimulate Axonal Sprouting Toward but Not beyond the Wound Edge. Molecular and cellular neurosciences. 2002;21(3):436–53. . 12498785 10.1006/mcne.2002.1185

[47] Isgor C, Pare C, McDole B, Coombs P, Guthrie K. Expansion of the dentate mossy fiber-CA3 projection in the brain-derived neurotrophic factor-enriched mouse hippocampus. Neuroscience. 2015;288:10–23. 10.1016/j.neuroscience.2014.12.036 25555929 PMC4324623

[48] Cadet JL, Krasnova IN. Molecular bases of methamphetamine-induced neurodegeneration. International review of neurobiology. 2009;88:101–19. 10.1016/S0074-7742(09)88005-7 . 19897076 PMC8247532

[49] Escubedo E, Guitart L, Sureda FX, Jimenez A, Pubill D, Pallas M, et al Microgliosis and down-regulation of adenosine transporter induced by methamphetamine in rats. Brain research. 1998;814(1–2):120–6. . 9838075 10.1016/s0006-8993(98)01065-8

[50] Kuhn DM, Francescutti-Verbeem DM, Thomas DM. Dopamine quinones activate microglia and induce a neurotoxic gene expression profile: relationship to methamphetamine-induced nerve ending damage. Annals of the New York Academy of Sciences. 2006;1074:31–41. 10.1196/annals.1369.003 . 17105901

[51] Polesskaya O, Silva J, Sanfilippo C, Desrosiers T, Sun A, Shen J, et al Methamphetamine causes sustained depression in cerebral blood flow. Brain research. 2011;1373:91–100. 10.1016/j.brainres.2010.12.017 21156163 PMC3026925

[52] Chung YA, Peterson BS, Yoon SJ, Cho SN, Chai S, Jeong J, et al In vivo evidence for long-term CNS toxicity, associated with chronic binge use of methamphetamine. Drug and alcohol dependence. 2010;111(1–2):155–60. 10.1016/j.drugalcdep.2010.04.005 . 20566251

[53] Agmon A, Connors BW. Thalamocortical responses of mouse somatosensory (barrel) cortex in vitro. Neuroscience. 1991;41(2–3):365–79. . 1870696 10.1016/0306-4522(91)90333-j

[54] Marshall JF, Belcher AM, Feinstein EM, O'Dell SJ. Methamphetamine-induced neural and cognitive changes in rodents. Addiction. 2007;102 Suppl 1:61–9. 10.1111/j.1360-0443.2006.01780.x . 17493054

[55] O'Dell SJ, Marshall JF. Effects of vibrissae removal on methamphetamine-induced damage to rat somatosensory cortical neurons. Synapse. 2002;43(2):122–30. 10.1002/syn.10016 . 11754491

[56] O'Dell SJ, Marshall JF. Neurotoxic regimens of methamphetamine induce persistent expression of phospho-c-Jun in somatosensory cortex and substantia nigra. Synapse. 2005;55(3):137–47. 10.1002/syn.20098 . 15549691

[57] Fujita S, Adachi K, Koshikawa N, Kobayashi M. Spatiotemporal dynamics of excitation in rat insular cortex: intrinsic corticocortical circuit regulates caudal-rostro excitatory propagation from the insular to frontal cortex. Neuroscience. 2010;165(1):278–92. 10.1016/j.neuroscience.2009.09.073 . 19800943

[58] Kobayashi M, Sasabe T, Shigihara Y, Tanaka M, Watanabe Y. Gustatory imagery reveals functional connectivity from the prefrontal to insular cortices traced with magnetoencephalography. PloS one. 2011;6(7):e21736 10.1371/journal.pone.0021736 21760903 PMC3132751

[59] Eckert MA, Menon V, Walczak A, Ahlstrom J, Denslow S, Horwitz A, et al At the heart of the ventral attention system: the right anterior insula. Human brain mapping. 2009;30(8):2530–41. 10.1002/hbm.20688 19072895 PMC2712290

[60] Taylor KS, Seminowicz DA, Davis KD. Two systems of resting state connectivity between the insula and cingulate cortex. Human brain mapping. 2009;30(9):2731–45. 10.1002/hbm.20705 . 19072897 PMC6871122

[61] Marks GA, Sachs OW, Birabil CG. Blockade of GABA, type A, receptors in the rat pontine reticular formation induces rapid eye movement sleep that is dependent upon the cholinergic system. Neuroscience. 2008;156(1):1–10. 10.1016/j.neuroscience.2008.06.067 18706488 PMC2614892

[62] Watson CJ, Lydic R, Baghdoyan HA. Sleep duration varies as a function of glutamate and GABA in rat pontine reticular formation. Journal of neurochemistry. 2011;118(4):571–80. 10.1111/j.1471-4159.2011.07350.x 21679185 PMC3144159

[63] Mitler MM. Evaluation of treatment with stimulants in narcolepsy. Sleep. 1994;17(8 Suppl):S103–6. . 7701190 10.1093/sleep/17.suppl_8.s103

[64] Mitler MM, Hajdukovic R, Erman MK. Treatment of narcolepsy with methamphetamine. Sleep. 1993;16(4):306–17. 8341891 PMC2267865

[65] Hoffman WF, Schwartz DL, Huckans MS, McFarland BH, Meiri G, Stevens AA, et al Cortical activation during delay discounting in abstinent methamphetamine dependent individuals. Psychopharmacology. 2008;201(2):183–93. 10.1007/s00213-008-1261-1 18685833 PMC2835463

[66] Krubitzer LPJ. Evolution of association pallial areas: parietal association areas in mammals. Encyclopedic Reference of Neuroscience 2008 p. 1225–31.

[67] Lu H, Zou Q, Gu H, Raichle ME, Stein EA, Yang Y. Rat brains also have a default mode network. Proceedings of the National Academy of Sciences of the United States of America. 2012;109(10):3979–84. 10.1073/pnas.1200506109 22355129 PMC3309754

[68] Pfefferbaum A, Chanraud S, Pitel AL, Muller-Oehring E, Shankaranarayanan A, Alsop DC, et al Cerebral blood flow in posterior cortical nodes of the default mode network decreases with task engagement but remains higher than in most brain regions. Cerebral cortex. 2011;21(1):233–44. 10.1093/cercor/bhq090 20484322 PMC3000573

[69] Raichle ME, Snyder AZ. A default mode of brain function: a brief history of an evolving idea. NeuroImage. 2007;37(4):1083–90; discussion 97–9. 10.1016/j.neuroimage.2007.02.041 . 17719799

[70] Huang YH, Tsai SJ, Su TW, Sim CB. Effects of repeated high-dose methamphetamine on local cerebral glucose utilization in rats. Neuropsychopharmacology: official publication of the American College of Neuropsychopharmacology. 1999;21(3):427–34. 10.1016/S0893-133X(99)00029-9 . 10457540

[71] Krettek JE, Price JL. Amygdaloid projections to subcortical structures within the basal forebrain and brainstem in the rat and cat. The Journal of comparative neurology. 1978;178(2):225–54. 10.1002/cne.901780204 . 627625

[72] Burrows KB, Meshul CK. High-dose methamphetamine treatment alters presynaptic GABA and glutamate immunoreactivity. Neuroscience. 1999;90(3):833–50. . 10218784 10.1016/s0306-4522(98)00506-5

[73] Royal Pharmaceutical Society of Great Britain. Martindale: the complete drug reference. London: Pharmaceutical Press; 1999.

[74] Hoogland IC, Houbolt C, van Westerloo DJ, van Gool WA, van de Beek D. Systemic inflammation and microglial activation: systematic review of animal experiments. Journal of neuroinflammation. 2015;12:114 10.1186/s12974-015-0332-6 26048578 PMC4470063

[75] Grobler SR, Chikte U, Westraat J. The pH Levels of Different Methamphetamine Drug Samples on the Street Market in Cape Town. ISRN dentistry. 2011;2011:974768 10.5402/2011/974768 21991491 PMC3189445

