# Correction: Chronic Methamphetamine Effects on Brain Structure and Function in Rats

**DOI:** 10.1371/journal.pone.0172080

**Published:** 2017-02-08

**Authors:** Panayotis K. Thanos, Ronald Kim, Foteini Delis, Mala Ananth, George Chachati, Mark J. Rocco, Ihssan Masad, Jose A. Muniz, Samuel C. Grant, Mark S. Gold, Jean Lud Cadet, Nora D. Volkow

The affiliation for the seventh author is incorrect. Ihssan Masad is affiliated with: Department of Biomedical Systems and Informatics Engineering, Hijjawi Faculty for Engineering Technology, Yarmouk University, Irbid 21163, Jordan.
